# 4-Chloro-3-fluoro-2-methyl­aniline–pyrrolidine-2,5-dione (1/1)

**DOI:** 10.1107/S1600536808018795

**Published:** 2008-06-28

**Authors:** Benjamin A. Mayes, Patrick McGarry, Adel Moussa, David J. Watkin

**Affiliations:** a Idenix Pharmaceuticals, 60 Hampshire Street, Cambridge, MA 02139, USA; b Department of Chemical Crystallography, Chemical Research Laboratory, University of Oxford, Mansfield Road, Oxford OX1 3TA, England

## Abstract

Chlorination of 3-fluoro-2-methyl­aniline with *N*-chloro­succinimide gave one major regioisomer whose structure was determined by X-ray crystallography. The product was found to have cocrystallized with succinimide, giving the title compound, C_7_H_7_ClFN·C_4_H_5_NO_2_. The crystal structure is stabilized by N—H⋯O hydrogen-bonding and π–π stacking inter­actions with a centroid–centroid distance of 3.4501 (8) Å.

## Related literature

For related literature, see: Lazar *et al.* (2004 ▶); Marterer *et al.* (2003 ▶); Nickson & Roche-Dolson (1985 ▶); Shapiro *et al.* (2006 ▶); Tukada & Mazaki (1997 ▶); Zanka & Kubota (1999 ▶); Görbitz (1999 ▶).
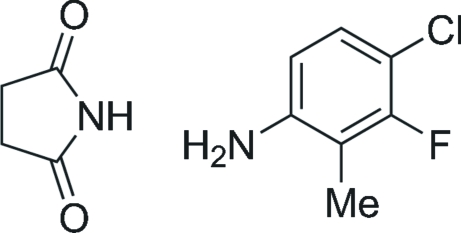


## Experimental

### 

#### Crystal data

C_7_H_7_ClFN·C_4_H_5_NO_2_
*M* *_r_* = 258.68
Triclinic, 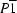

*a* = 7.3853 (2) Å
*b* = 7.4390 (2) Å
*c* = 11.5571 (4) Å
α = 73.1036 (13)°
β = 85.9336 (12)°
γ = 71.3703 (14)°
*V* = 575.53 (3) Å^3^
*Z* = 2
Mo *K*α radiation
μ = 0.34 mm^−1^
*T* = 120 K
0.75 × 0.44 × 0.41 mm

#### Data collection

Nonius KappaCCD diffractometer
Absorption correction: multi-scan (*DENZO*/*SCALEPACK*; Otwinowski & Minor, 1997 ▶) *T*_min_ = 0.47, *T*_max_ = 0.87
16689 measured reflections
2904 independent reflections
2610 reflections with *I* > 2σ(*I*)
*R*_int_ = 0.033

#### Refinement

*R*[*F* ^2^ > 2σ(*F* ^2^)] = 0.032
*wR*(*F* ^2^) = 0.087
*S* = 0.88
2904 reflections
154 parameters
H-atom parameters constrained
Δρ_max_ = 0.38 e Å^−3^
Δρ_min_ = −0.37 e Å^−3^

### 

Data collection: *COLLECT* (Nonius, 2001 ▶); cell refinement: *DENZO*/*SCALEPACK* (Otwinowski & Minor, 1997 ▶); data reduction: *DENZO*/*SCALEPACK*; program(s) used to solve structure: *SIR92* (Altomare *et al.*, 1994 ▶); program(s) used to refine structure: *CRYSTALS* (Betteridge *et al.*, 2003 ▶); molecular graphics: *CAMERON* (Watkin *et al.*, 1996 ▶); software used to prepare material for publication: *CRYSTALS*.

## Supplementary Material

Crystal structure: contains datablocks I, global. DOI: 10.1107/S1600536808018795/lh2642sup1.cif

Structure factors: contains datablocks I. DOI: 10.1107/S1600536808018795/lh2642Isup2.hkl

Additional supplementary materials:  crystallographic information; 3D view; checkCIF report

## Figures and Tables

**Table 1:** Hydrogen-bond geometry (Å, °)

*D*—H⋯*A*	*D*—H	H⋯*A*	*D*⋯*A*	*D*—H⋯*A*
N12—H1⋯O16^i^	0.85	2.11	2.945 (2)	168
N8—H9⋯O16^i^	0.84	2.18	2.915 (2)	147
N8—H11⋯O17	0.88	2.17	3.030 (2)	166

Symmetry code: (i) 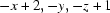.

**Fig. 1.**
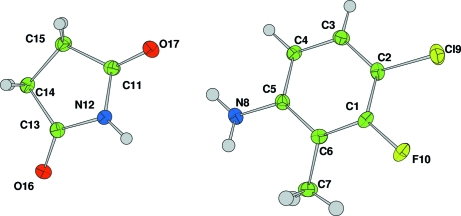
The title compound with displacement ellipsoids drawn at the 50% probability level. H atoms are shown as spheres of arbitary radius.

**Fig. 2.**
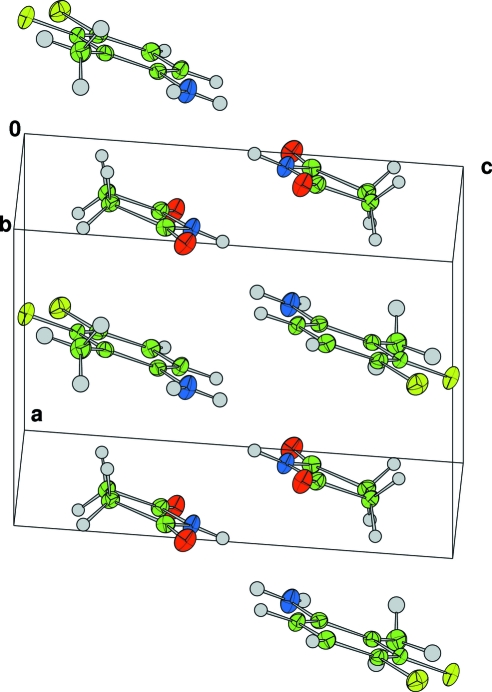
Plane-to-plane stacking of alternate molecules parallel to the a axis.

**Fig. 3.**
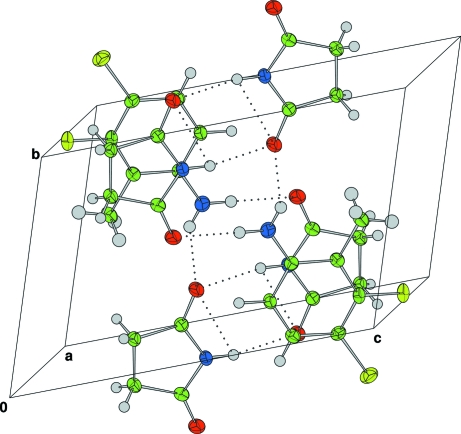
The hydrogen bonds (dotted lines) in the π -π stacks.

**Fig. 4.**
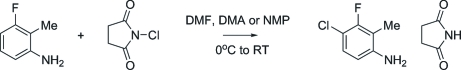
Synthetic scheme.

## supplementary crystallographic information

### Comment

Chlorination of anilines with *N*-chlorosuccinimide (NCS) can provide access to poly-substituted aromatic compounds, useful as high-value synthetic intermediates (Lazar *et al., *2004; Marterer *et al.*, 2003; Nickson & Roche-Dolson, 1985; Shapiro *et al.*, 2006; Zanka & Kubota, 1999). In the present example, treatment of 3-fluoro-2-methylaniline with NCS in polar solvents (*e.g. N*,*N*-dimethylformamide) resulted in chlorination *para* to the NH_2 _as the primary regioisomer in 10-fold excess relative to the undesired *ortho* isomer.

The sample was supplied in the form of large crystalline aggregates (4 mm across) coated with perfluoropolyether oil as a preservative. A large (0.8x0.8x0.4 mm) section was cut from the mass. The material did not have a strong cleavage - the crystals just fractured erratically. Because of the risk that further cutting might totally destroy the sample, an initial X-ray data set was measured from this large sample. The results confirmed the expected structure, but also showed a co-crystallized molecule of succinimide (Tukada & Mazaki, 1997).

At the end of the initial data collection, the sample was further subdivided into an irregular block approximately 0.41x0.44x0.75 mm. Prescans showed that the further cutting of the crystal had introduced fractures, but the sample was still amenable to analysis. Because of the degraded quality of the crystal, a data set with a target redundancy of 3 (as opposed to the usual 1) was collected. This highly redundant dataset would enable corrections to be made for the poor crystal quality.

Structure solution was slightly complicated because of the unexpected succinimide, but after that refinement and the location of all hydrogen atoms was normal. The two components are shown in Fig. 1. Fig. 2 shows the plane-to-plane alternate stacking of the components, with minimum inter-planar spacing of 3.37Å - presumable π - π stacking. The columns of molecules are interconnected by N-H···O hydrogen bonds which form discreet centrosymmetric 4-component clusters (Fig. 3).

### Experimental

3-Fluoro-2-methylaniline (550 mg, 4.40 mmol) was dissolved in *N*,*N*-dimethylformamide (DMF), *N*,*N*-dimethylacetamide (DMA) or 1-methyl-2-pyrrolidinone (NMP) (5 ml) and cooled to 0–5°C under argon. *N*-Chlorosuccinimide (586 mg, 4.39 mmol) was added and the mixture was allowed to warm to room temperature over 15 h (Fig. 4). Dilution with ethyl acetate, washing with water, drying (sodium sulfate), filtration and evaporation of the solvents gave a crude oil.

Crystals were grown from isopropyl ether by seeding and storing at 4°C for two weeks. The solvent was decanted and the crystals coated with 2 drops of FOMBLIN perfluoropolyether oil.

Additional methods of characterization were recorded: m.p. 75.5–76.0°C; ^1^H (400 MHz, d3-MeCN): δ = 2.04 (3*H*, d, *J* 2.0 Hz, C*H*_3_), 2.62 (4*H*, s, C*H*_2_C*H*_2_), 4.32 (2*H*, br-s, N*H*_2_), 6.46 (1*H*, dd, *J* 8.6 Hz, *J* 0.8 Hz), 7.00 (1*H*, a-t, *J* 8.6 Hz), 8.83 (1*H*, br-s, N*H*). ^13^C (100 MHz, d3-MeCN): δ = 9.12, 9.18 (*C*H_3_), 30.26 (*C*H_2_*C*H_2_), 107.99, 108.19 (*C*-2), 111.16 (*C*-6), 111.34, 111.37 (*C*-4), 127.97, 127.98 (*C*-3), 147.76, 147.82 (*C*-5), 156.09, 158.47 (*C*-1), 179.33 (2 *xC*=O) (using crystallographic numbering).

### Refinement

The relatively large ratio of minimum to maximum corrections applied in the multiscan process (1:1.85) reflect the poor quality of the sample.

Difficulties in selecting an integration box suitable for all frames were taken into account (Görbitz, 1999) by the multi-scan inter-frame scaling (*DENZO*/*SCALEPACK*, Otwinowski & Minor, 1997).

The H atoms were all located in a difference map, but those attached to carbon atoms were repositioned geometrically. The H atoms were initially refined with soft restraints on the bond lengths and angles to regularize their geometry (C—H in the range 0.93–0.98, N—H in the range 0.86–0.89 N—H to 0.86 Å) and *U*_iso_(H) (in the range 1.2–1.5 times *U*_eq_ of the parent atom), after which the positions were refined with riding constraints.

### Crystal data

**Table:**

C_7_H_7_ClFN·C_4_H_5_NO_2_	*Z* = 2
*M**_r_* = 258.68	*F*_000_ = 268
Triclinic, *P*1	*D*_x_ = 1.493 Mg m^−^^3^
*a* = 7.3853 (2) Å	Mo *K*α radiation λ = 0.71073 Å
*b* = 7.4390 (2) Å	Cell parameters from 2870 reflections
*c* = 11.5571 (4) Å	θ = 5–29º
α = 73.1036 (13)º	µ = 0.34 mm^−^^1^
β = 85.9336 (12)º	*T* = 120 K
γ = 71.3703 (14)º	Plate, colourless
*V* = 575.53 (3) Å^3^	0.75 × 0.44 × 0.41 mm

### Data collection

**Table:**

Nonius KappaCCD diffractometer	2610 reflections with *I* > 2σ(*I*)
Monochromator: graphite	*R*_int_ = 0.033
*T* = 120 K	θ_max_ = 28.7º
ω scans	θ_min_ = 5.4º
Absorption correction: multi-scan(DENZO/SCALEPACK; Otwinowski & Minor, 1997)	*h* = −9→9
*T*_min_ = 0.47, *T*_max_ = 0.87	*k* = −10→9
16689 measured reflections	*l* = −15→15
2904 independent reflections	

### Refinement

**Table:**

Refinement on *F*^2^	Hydrogen site location: inferred from neighbouring sites
Least-squares matrix: full	H-atom parameters constrained
*R*[*F*^2^ > 2σ(*F*^2^)] = 0.032	Method = Modified Sheldrick *w* = 1/[σ^2^(*F*^2^) + (0.05*P*)^2^ + 0.35*P*], where *P* = [max(*F*_o_^2^,0) + 2*F*_c_^2^]/3
*wR*(*F*^2^) = 0.087	(Δ/σ)_max_ = 0.001
*S* = 0.88	Δρ_max_ = 0.38 e Å^−^^3^
2904 reflections	Δρ_min_ = −0.37 e Å^−^^3^
154 parameters	Extinction correction: None
Primary atom site location: structure-invariant direct methods	

### Fractional atomic coordinates and isotropic or equivalent isotropic displacement parameters (Å^2^)

**Table:**

	*x*	*y*	*z*	*U*_iso_*/*U*_eq_	
C11	0.94307 (16)	0.30918 (17)	0.66142 (11)	0.0207	
N12	0.99166 (15)	0.16670 (15)	0.59934 (9)	0.0223	
C13	1.10284 (16)	−0.01507 (17)	0.66627 (11)	0.0205	
C14	1.14595 (16)	−0.00013 (17)	0.78815 (10)	0.0204	
C15	1.04097 (16)	0.21516 (17)	0.78494 (11)	0.0211	
O16	1.15499 (13)	−0.16202 (13)	0.62979 (8)	0.0275	
O17	0.83954 (13)	0.47656 (13)	0.62086 (8)	0.0280	
H141	1.2797	−0.0325	0.7987	0.0245*	
H142	1.0987	−0.0916	0.8511	0.0240*	
H151	1.1221	0.2859	0.7939	0.0258*	
H152	0.9471	0.2271	0.8474	0.0263*	
H1	0.9510	0.1824	0.5287	0.0265*	
C1	0.34402 (16)	0.96277 (18)	0.14329 (10)	0.0201	
C2	0.28607 (16)	1.11710 (17)	0.19551 (11)	0.0207	
C3	0.34232 (16)	1.08204 (17)	0.31427 (11)	0.0217	
C4	0.45586 (16)	0.89539 (17)	0.37673 (10)	0.0210	
C5	0.51642 (16)	0.74038 (17)	0.32214 (10)	0.0188	
C6	0.45694 (16)	0.77380 (17)	0.20237 (10)	0.0194	
C7	0.51289 (19)	0.60873 (19)	0.14308 (12)	0.0269	
N8	0.63360 (16)	0.55949 (16)	0.38422 (10)	0.0277	
Cl9	0.14163 (4)	1.34735 (4)	0.11329 (3)	0.0293	
F10	0.28435 (11)	0.99849 (12)	0.02806 (6)	0.0291	
H31	0.3012	1.1851	0.3475	0.0277*	
H41	0.4953	0.8705	0.4581	0.0257*	
H71	0.6503	0.5518	0.1414	0.0420*	
H72	0.4641	0.5027	0.1863	0.0416*	
H73	0.4639	0.6536	0.0618	0.0429*	
H9	0.6698	0.4664	0.3519	0.0324*	
H11	0.6736	0.5392	0.4585	0.0317*	

### Atomic displacement parameters (Å^2^)

**Table:**

	*U*^11^	*U*^22^	*U*^33^	*U*^12^	*U*^13^	*U*^23^
C11	0.0199 (5)	0.0202 (5)	0.0235 (6)	−0.0059 (4)	0.0007 (4)	−0.0088 (4)
N12	0.0264 (5)	0.0201 (5)	0.0187 (5)	−0.0024 (4)	−0.0048 (4)	−0.0072 (4)
C13	0.0196 (5)	0.0201 (5)	0.0209 (5)	−0.0039 (4)	−0.0013 (4)	−0.0064 (4)
C14	0.0196 (5)	0.0216 (5)	0.0195 (5)	−0.0049 (4)	−0.0029 (4)	−0.0061 (4)
C15	0.0198 (5)	0.0232 (5)	0.0221 (6)	−0.0066 (4)	−0.0014 (4)	−0.0090 (4)
O16	0.0325 (5)	0.0212 (4)	0.0259 (5)	0.0005 (4)	−0.0056 (4)	−0.0108 (4)
O17	0.0302 (5)	0.0206 (4)	0.0305 (5)	−0.0012 (3)	−0.0058 (4)	−0.0090 (4)
C1	0.0196 (5)	0.0262 (6)	0.0156 (5)	−0.0089 (4)	−0.0015 (4)	−0.0051 (4)
C2	0.0180 (5)	0.0187 (5)	0.0228 (6)	−0.0049 (4)	−0.0023 (4)	−0.0023 (4)
C3	0.0217 (5)	0.0206 (5)	0.0237 (6)	−0.0051 (4)	0.0005 (4)	−0.0094 (4)
C4	0.0213 (5)	0.0228 (5)	0.0184 (5)	−0.0043 (4)	−0.0026 (4)	−0.0074 (4)
C5	0.0165 (5)	0.0190 (5)	0.0196 (5)	−0.0041 (4)	0.0003 (4)	−0.0050 (4)
C6	0.0181 (5)	0.0218 (5)	0.0205 (5)	−0.0081 (4)	0.0020 (4)	−0.0077 (4)
C7	0.0290 (6)	0.0277 (6)	0.0276 (6)	−0.0077 (5)	0.0010 (5)	−0.0144 (5)
N8	0.0317 (6)	0.0207 (5)	0.0241 (5)	0.0027 (4)	−0.0051 (4)	−0.0071 (4)
Cl9	0.02920 (17)	0.02088 (16)	0.03136 (17)	−0.00375 (11)	−0.00674 (12)	−0.00040 (11)
F10	0.0341 (4)	0.0346 (4)	0.0175 (3)	−0.0092 (3)	−0.0072 (3)	−0.0055 (3)

### Geometric parameters (Å, °)

**Table:**

C11—N12	1.3890 (14)	C2—C3	1.3885 (17)
C11—C15	1.5141 (16)	C2—Cl9	1.7335 (12)
C11—O17	1.2075 (14)	C3—C4	1.3826 (16)
N12—C13	1.3689 (15)	C3—H31	0.913
N12—H1	0.852	C4—C5	1.4075 (16)
C13—C14	1.5082 (16)	C4—H41	0.952
C13—O16	1.2235 (14)	C5—C6	1.4096 (16)
C14—C15	1.5309 (16)	C5—N8	1.3629 (14)
C14—H141	0.947	C6—C7	1.5076 (16)
C14—H142	0.970	C7—H71	0.968
C15—H151	0.943	C7—H72	0.963
C15—H152	0.968	C7—H73	0.955
C1—C2	1.3848 (17)	N8—H9	0.842
C1—C6	1.3826 (16)	N8—H11	0.882
C1—F10	1.3569 (13)		
			
N12—C11—C15	107.80 (9)	C1—C2—C3	118.84 (11)
N12—C11—O17	124.27 (11)	C1—C2—Cl9	119.74 (9)
C15—C11—O17	127.93 (11)	C3—C2—Cl9	121.41 (9)
C11—N12—C13	113.60 (10)	C2—C3—C4	119.37 (11)
C11—N12—H1	125.7	C2—C3—H31	117.6
C13—N12—H1	120.5	C4—C3—H31	123.1
N12—C13—C14	108.74 (9)	C3—C4—C5	121.32 (11)
N12—C13—O16	123.80 (11)	C3—C4—H41	119.8
C14—C13—O16	127.46 (11)	C5—C4—H41	118.9
C13—C14—C15	104.82 (9)	C4—C5—C6	119.65 (11)
C13—C14—H141	109.5	C4—C5—N8	120.21 (11)
C15—C14—H141	111.9	C6—C5—N8	120.13 (10)
C13—C14—H142	109.2	C5—C6—C1	117.02 (10)
C15—C14—H142	111.7	C5—C6—C7	120.92 (11)
H141—C14—H142	109.5	C1—C6—C7	122.05 (11)
C14—C15—C11	104.97 (9)	C6—C7—H71	111.7
C14—C15—H151	114.0	C6—C7—H72	111.2
C11—C15—H151	109.0	H71—C7—H72	106.7
C14—C15—H152	112.8	C6—C7—H73	111.5
C11—C15—H152	109.9	H71—C7—H73	108.1
H151—C15—H152	106.2	H72—C7—H73	107.4
C2—C1—C6	123.78 (11)	C5—N8—H9	120.4
C2—C1—F10	118.07 (10)	C5—N8—H11	120.3
C6—C1—F10	118.14 (10)	H9—N8—H11	119.3

### Hydrogen-bond geometry (Å, °)

**Table:**

*D*—H···*A*	*D*—H	H···*A*	*D*···*A*	*D*—H···*A*
N12—H1···O16^i^	0.85	2.11	2.945 (2)	168
N8—H9···O16^i^	0.84	2.18	2.915 (2)	147
N8—H11···O17	0.88	2.17	3.030 (2)	166

Symmetry codes: (i) −*x*+2, −*y*, −*z*+1.
